# Design of colorimetric nanostructured sensor phases for simple and fast quantification of low concentrations of acid vapors

**DOI:** 10.1007/s00604-023-05723-0

**Published:** 2023-03-27

**Authors:** M. D. Fernández-Ramos, M. Bastida-Armesto, R. Blanc-García, L. F. Capitán-Vallvey, A. L. Medina-Castillo

**Affiliations:** 1 ECsens, Department of Analytical Chemistry, University of Granada, 18071 Granada, Spain; 2 Unit of Excellence in Chemistry Applied to Biomedicine and the Environment of the University of Granada, Granada, Spain; 3 Department of Analytical Chemistry, University of Granada, 18071 Granada, Spain

**Keywords:** Color nanostructured membranes, Acid vapors, Nanostructured sensing phases, PANI, Cultural heritage, Indoor environments

## Abstract

Two colorimetric nanostructured sensor phases (Color-NSPs) for the determination of low concentrations of acid vapors in the atmosphere of paper storage rooms have been designed and characterized. The acid vapor determination is based on the color change that occurs in polyaniline (PANI) in the presence of acids when it goes from its emeraldine base form (blue) to its emeraldine salt form (green). To synthesize the Color-NSPs, two methods have been used, a one-step method performed by grafting polyaniline onto a cellulose membrane (Cellu-PANI) and a two-step method in which in the first step, polyaniline is grafted onto the surface of polymeric nanoparticles (NPs-PANI), and in a second step, NPs-PANI are immobilized into the pores of a nylon membrane (Nylon-NPs PANI). The response of the sensors versus acid vapor was measured by color coordinates with a photographic camera. A linear response range from 1 ppmv to 7 ppmv was found for both sensors, and the detection limits were 0.95 ppmv (1.2 % RSD) and 0.40 ppmv (0.8 % RSD) for Cellu-PANI and Nylon-NPs PANI, respectively. In addition, both sensors showed complete reversibility and a short exposition time (5 min). The potential applicability of the Color-NSPs in the control of the exposure of paper heritage collections to outdoor- and indoor-generated gaseous pollutants was demonstrated by determining acid vapors in museums. The method was validated with an external reference method; the paired test was applied, and *p-*values greater than 5% were obtained, indicating an excellent correlation and showing that the Color-NSPs reported are simple, fast, and an economical alternative to control and protect cultural heritage materials in indoor environments.

**Graphical Abstract:**

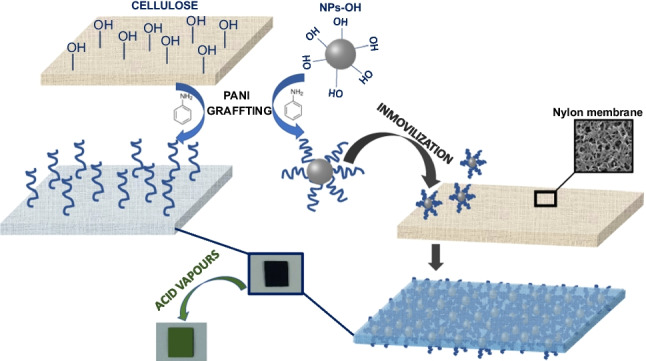

**Supplementary information:**

The online version contains supplementary material available at 10.1007/s00604-023-05723-0.

## Introduction

Cultural heritage is one of the economic pillars of a country, not only for the cultural interest it presents, but also as an economical asset, so its long-term protection is highly demanding. When cultural heritage objects, as well as the materials used for their restoration, are degraded, these can emit volatile organic acids mainly acetic and/or formic. This acidic environment causes the deterioration of other cultural heritage objects, altering them visibly or not visibly at the molecular level [1, 2]. Organic acids are of internal origin emitted mainly by oxidative and hydrolytic reactions in displayed or stored objects made of wood, paper, and some plastic [3]. They have been found in indoor environments at concentrations between 9 and 88 ppb [4]. The *ASHRAE Handbook* [5] states that the maximum average acid vapor concentration for the 1- or 100-year conservation objective for museums, galleries, libraries, and archival collections is 163 ppmv and 16 ppmv, respectively, although these values may be lower depending on the type of material. Organic acid vapors can cause corrosion of metals and degradation of calcareous materials such as limestone, ceramics, and fossils as well as a reduction in the degree of polymerization of cellulose acetate objects, and over time, this corrosion can lead to the total deterioration of the object [6].

Therefore, to carry out preventive actions on human health and the conservation of cultural assets, precise and low-cost methods are required that allow constant and immediate monitoring of these acids. The most widely used methodology to determine the concentration of acid vapors in the air is based on the use of diffusion tubes, passive samplers, and dosimeters. But these methods require a long exposure time (normally 1 or 2 months), the use of sophisticated measuring techniques (e.g., gas chromatography/mass spectrometry), and thus highly qualified staff; consequently, they are expensive and are not routinely used [1, 6]. As an alternative, analytical methods based on polyaniline quartz crystal microbalance have been proposed, although the microbalance signals are irreversible, imprecise, and the instrument needs frequent recalibration [7, 8].

Chemical sensors are an alternative to evaluate the environmental quality and ensure the conservation of cultural heritage because they are simple, inexpensive, nontoxic, totally recyclable, and can be used by non-specialized staff. Smart sensors are continuously required in the cultural heritage and are usually based on nanomaterials that present high selectivity and specificity for the recognition of specific compounds; the most interesting in this area are electrochemical sensors, optical sensors based on surface enhanced Raman scattering (SERS) [9], fiber optics [10], and colorimetric sensors [11].

The simplest, oldest, and most widely used optical pH sensor is the conventional A-D (acid detector) color strip. These strips were originally developed to measure the “vinegar syndrome” [12] and are based on an acid indicator that changes color. They offer a useful and quick visual guide of expected levels of volatile acids. The strips have an exposure time of only 1 or 2 days at room temperature, depending on humidity conditions, providing an economic alternative. The main disadvantage of A-D strips is that they do not provide direct readings of vapor concentration in the air. They only provide approximate values by establishing a range of concentration using a color chart. Today A-D strips are commercially available. In the last decades, more sophisticated pH sensors with different transduction have emerged. As an alternative to the A-D strips, optical sensors based on the retention of conventional pH indicators in sol–gel matrices have been developed [13]. These sensors can detect and quantitatively determine the pH in the air only in the range of pH 5–8, but their exposition time is estimated at 24 h.

Chemical sensors based on conducting polymer as polyaniline (PANI) [14] are very attractive due to their exclusive electrical characteristics, environmental stability, and easy fabrication process. On the other hand, PANI has the advantage that can act as both polymeric support and a transduction system. A large number of room-temperature electrochemical gas sensors based on PANI on different supports (glass, PET, etc.) can be found in the literature [15–18]. The PANI films present a pH-dependent absorption spectrum in the visible region with different forms: emeraldine salt (protonated), which is green; emeraldine base (partially protonated) which is blue, and pernigraniline (deprotonate) of purple. These forms are related to pH, which leads to a reversible response over the full pH range [19]. Polyaniline-functionalized nanofibers have also been used for colorimetric and electrochemical detection of hydrogen chloride and ammonia vapor respectively with promising results [20, 21]. pH sensors based on metal oxides (mainly ZnO) doped with PANI have also been reported [22]. By using ZnO, the performance of the sensors is enhanced due to an electronic effect, although the agglomeration of metal oxide would limit the further improvement of the sensing properties. Recently, photonic crystal hydrogel pH sensors that rely on diffraction-induced color change have also been developed, enabling a naked-eye detection by color changes [23, 24].

In this study, two colorimetric nanostructured sensor phases (Color-NSPs) for the detection of low concentrations of acid vapors have been designed and characterized. The Color-NSPs studied were reported synthesized using two different methodologies: a one-step method based on grafting polyaniline onto a cellulose membrane and a two-step method in which polyaniline is first grafted onto the surface of polymeric nanoparticles (NPs-PANI) and then immobilized in the pores of a nylon membrane with a pore diameter of 450 nm.

The Color-NSPs allow the quick, reversible, and easy determination of the concentration of acid vapor with a low exposure time. The Color-NSPs were successfully applied to exposure control of paper heritage collections to outdoor- and indoor-generated acid vapors. They were validated with an external reference method [12], providing a *p*-value greater than 5%, indicating an excellent correlation, and demonstrating that they are simple, fast, and economical alternatives to control and protect cultural heritage property in indoor environments.

## Experimental section

### Reagents and materials

Acetic acid 99.7%, formic acid 98%, aniline, ammonium persulfate, ammonia, hydrochloric acid, N-methyl-2-pyrrolidone, calcium carbonate, disodium hydrogen phosphate anhydrous, divinyl sulfone (DVS), ethylene diamine, methanol, and all reagents came from Sigma-Aldrich Merck (Madrid, Spain). Cellulose filter paper from Filter-Lab (www.fanoia.com) (Barcelona, Spain), ref. 1238 (basis weight 80 g m^−2^; thickness 150 μm; retention 20–25 μm), and nanocrystalWhatman®NYtran (SPC) nylon blotting membranes, w x L 11 cm × 14 cm, pore size 0.45 μm, pkg of 10 ea from Sigma Aldrich Merck (Madrid, Spain). A-D strips from Image Permanence Institute (Rochester, NY, USA) were used as a reference method. All aqueous solutions were made using reverse osmosis-type quality water (Milli-RO 12 plus Milli-Q station from Millipore, conductivity 18.2 MΩ·cm).

### Instrumentation

A homemade climate chamber made of an opaque glass block, 4.4 cm high, 1.2 cm wide, and 3.0 cm long with an upper inlet for steam and a small hole in the bottom to prevent overpressure was used as a cell holder to put the sensor phases in contact with acid vapors (see Fig. S1). Through the climatic chamber, using an inlet pipe, the sensor phase is put in contact with acid vapors. To produce acid vapor atmospheres, acetic acid solution 3.0 mM aqueous prepared by dilution of a commercial HAc solution (99.7%) in pure water was vaporized using a controlled evaporator mixer system (CEM). It consists of a mass flow controller for measurement and control of carrier gas flow (synthetic air) and a mass flow meter for liquids (MiniCoriflow). The mixing of the liquid with the carrier gas flow is controlled by a 3-way EMC mixing valve. The acetic acid vapor was generated by dilution of an aqueous solution of 3 mM (0.3–3.0 g·h^−1^), with variable amounts of pure air (at 20 °C and 5 ln·min^−1^). In order to prepare, temperature-controlled heat exchanger was used to ensure complete evaporation of the liquid (118 °C for acetic acid). In addition, the system also enables the control of relative humidity (RH%) between 0 and 100%. All the experiments were replicated three times in order to evaluate the error ($\frac{st}{\sqrt{n}})$ where *s* is the standard deviation, *t* is the student t, and *n* is the number of replicates.

### Image capture and processing

The membrane sensing was imaged using a Canon Powershot G12 digital camera (Japan) placed inside of a homemade wooden enclosure [25] illuminated with two LED lamps (4.6 W, 6000 K, illumination inside of the box = 9680 Lx) placed at 45° with respect to the digital camera to minimize any interference from external light. The optimized settings used to photograph the sensing membrane were ISO 80; F 5.6; shutter speed 1/1600 s; aperture value f/8; focal distance 11 mm; white balance, automatic; resolution, 3648 × 2432; and mode, macro. To evaluate the color change, a photograph was taken in JPEG format. RGB and color coordinates were obtained from the region of interest (ROI) of the digitized membrane using ImageJ software (National Institutes of Health). The analytical parameter used is the difference between color coordinates before (control) and after the reaction with the analyte gas (CC_f_-CC_0_).

### Preparation of Color-NSPs by grafting polyaniline onto a cellulose membrane

Graft polymerization of polyaniline is initiated from amine groups. Thus, cellulose membranes were previously functionalized with amine groups.

#### Functionalization of cellulose with amine groups (Cellu-NH_2_)

First, the hydroxyl groups of cellulose were functionalized with vinyl sulfone groups to obtain Cellu-VS. To do so, a piece of cellulose (16 × 11 cm) was introduced into 70 mL of a solution of DVS (0.33 M) in sodium carbonate buffer (333 mM) at pH = 12.00 for 2 h. Subsequently, Cellu-VS was washed three times with distilled water for 15 min and dried at 50 °C in a vacuum oven. It is well-known that vinyl sulfone groups can react easily with amine groups in mild conditions by a Michael-type reaction [26]. Thus, in a second step, Cellu-VS (16 × 11 cm) was introduced into 70 mL of a solution of ethylene diamine (0.33 M) in phosphate buffer (100 mM) at pH = 8 for 4 h. Then, Cellu-NH_2_ was washed three times with distilled water and dried at 50 °C in a vacuum oven.

#### Grafting of polyaniline on Cellu-NH_2_

Polyaniline was grafted on the Cellu-NH_2_ via chemical oxidative polymerization at 0 °C. *Cellu-NH*_*2*_ (5 × 5 cm) was placed into 10 ml of aniline hydrochloride (0.15 M) dissolved in HCl (1.2 M). Then 10 ml of ammonium persulfate (0.15 Min 1.2 M HCl) was added dropwise with constant stirring, and the reaction proceeded on ice with constant stirring for 2 h. Subsequently, Cellu-PANI was placed in 0.1 M ammonia and sonicated for 30 min to convert polyaniline into emeraldine base form. Since the emeraldine base form is soluble in NMP, Cellu-PANI was washed with NMP to remove the PANI not covalently attached until the solvent remained colorless after sonication. Finally, Cellu-PANI was rinsed with purified water and dried at room temperature.

### Preparation of Color-NSPs by grafting polyaniline on the surface of polymeric nanoparticles

#### Synthesis of polymeric nanoparticles functionalized with hydroxyl groups

Hydroxylated polymeric nanoparticles (NPs-OH; 230 nm of diameter) were synthesized according to our previous paper [27]. Then NPs-OH were grafted with polyaniline following the same steps described above for the functionalization of Cellu-OH. Subsequently, NPs-PANI was dispersed in NMP, and the suspension was vacuum filtered using a nylon membrane with 450 nm of pore diameter. The NPs-PANI suspension was passed through the nylon membrane until the pores of the membrane were completely saturated with NPs-PANI. Then Nylon-NPs-PANI membrane was rinsed with deionized water and dried at room temperature.

## Results and discussion

### Fabrication of Color-NSPs

**Fig. 1:**
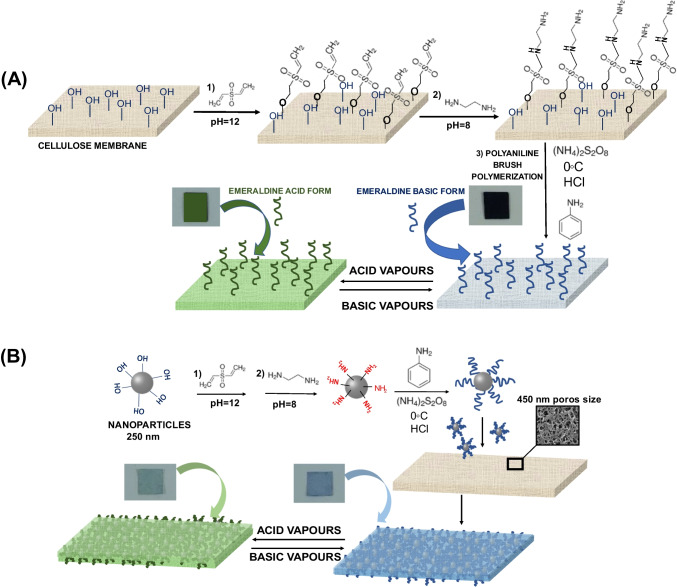
Grafting of polyaniline on cellulose membrane (**A**); grafting of polyaniline on the surface of polymeric nanoparticles and subsequent immobilization of nanoparticles into the porous nylon membrane (**B**)

The Color-NSPs reported in this work were synthesized using two different methodologies: (1) one-step method based on polyaniline grafting on a cellulose membrane and (2) two-step method in which in the first step, polyaniline is grafted on the surface of polymeric nanoparticles (NPs-PANI), and in a second step, NPs-PANI is immobilized into the pores of a nylon membrane with a pore diameter of 450 nm. The chemical structures of all materials and fabrication procedure of the Color-NSPs, Cellu-PANI and Nylon-NPs-PANI, are shown in Fig. 1. As can be seen, in both cases, a reversible color change is observed when the membranes are exposed to acid and basic vapors. The color changes from green (protonated form of emeraldine) to blue when the membranes are exposed to basic vapors (emeraldine base; deprotonated form).

### Study of the analytical parameters of Color-NSPs

The analytical parameters of Color-NSPs were selected by exposing the membranes at different concentrations of acid vapors for 5 min. Photographs were done before (controls) and after the exposure of the membranes to acid vapors. Then, the RGB and HSV color space coordinates were obtained and studied (see Fig. S2). As can be seen, the response of H, S, and V coordinates is lower than R, G, and B coordinates. On the other hand, Fig. S3 shows how the gray coordinate responds to a greater range of concentrations than R and B coordinates. Therefore, gray coordinate was selected as analytical signal to quantify the acid vapor concentration. In order to know the optimum exposure time, the Color-NSPs were exposed at different concentrations of acid vapors until a stable signal was reached. Fig. S4 shows that the maximum response is reached at 5 min.

### Analytical characterization of Color-NSPs

Color-NSPs were exposed at different concentrations of acid vapors (from 1 to 7 ppmv) at 25 °C and 55% RH (see Calibration plots Fig S5). The detection and quantification limits were calculated according to the methodology established by IUPAC [28], LOD = *t*_0_ + 3*s*_0_ and LOQ = *t*_0_ -10 *s*_0_, where *t*_0_ is the average blank signal (Color-NSPs without contact with dry air as blank) and *s*_0_ is the critical level or standard deviation of the blank, which was determined from eight replicate measurements.

Table 1 shows the analytical parameters of Color-NSPs. The precision in the measurement was evaluated in ten membranes (*n* = 3) at two concentration levels of acid vapors: 1 ppmv and 5 ppmv. In all cases, the relative standard deviation (RSD) was smaller than 5% (see Table 1). It is noteworthy that Nylon-NPs-PANI membranes are more accurate than Cellu-PANI.

**Table 1:** Characteristics of Color-NPs membranes for acid vapors

Analytical parameter	Color-NSPs	
	Cellu-PANI	Nylon-NPs-PANI
Measurement range (ppmv)	1.0–7.0	1.0–7.0
Slope (b)	0.60 ± 0.05	0.180 ± 0.001
Intercept (a)	0.060 ± 0.002	0.038 ± 0.002
LOD (ppmv)	0.95	0.40
LQD (ppmv)	3.18	1.56
Precision 1.0/5.0 ppmv (%RSD)	4.10/4.30	3.60/3.20
Response time (min)	5	
Lifetime (days)	At least 200	

**Fig. 2:**
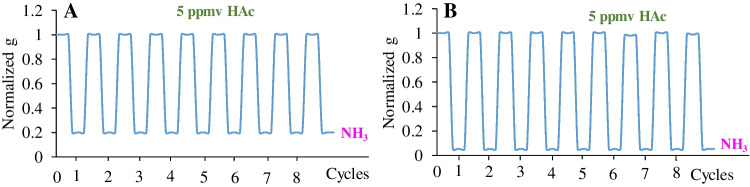
Activation-regeneration cycles of Color-NSPs. **A** Cellulose-PANI; **B** Nylon-NPs-PANI

The short-term precision (repeatability) was analyzed by recording acid vapor measurements three times for each experimental condition. To check the repeatability, the Color-NSPs were exposed to an atmosphere of 5 ppmv (see “Instrumentation” section) for 5 min. Next, Color-NSPs were reset from green to blue by incubating them into a solution of 0.1 M of ammonia for 30 s to recover the initial signal. Figure 2 shows the alternation of eight cycles based on the exposure of the membranes to acid vapors, following their immersion in ammonia solutions for 30 s. Then the membranes were left at room temperature until completely dry, and a repeatability of 0.5% for both Color-NSPs was obtained. As can be seen in Fig. 2, all the membranes support at least 8 activation-regeneration cycles.

Long-term precision was studied by measuring the response of the sensor when it is exposed to 5 ppmv of acid vapors for 120 days (the sensors were kept at room temperature in the dark). Long-term stability was done for a set of sensors using a Shewhart diagram. Long-term precision (T1) was defined as the signal that remain constant within the control line on the Shewhart chart. In addition, long-term precision (T2) was defined as the time during which the sensor responds to acid vapors, although it must be recalibrated. Fig. S6 shows the control chart for the sensor. As can be seen after 120 days, the sensor signal remains within the established control limits, showing excellent long-term stability.

**Fig. 3:**
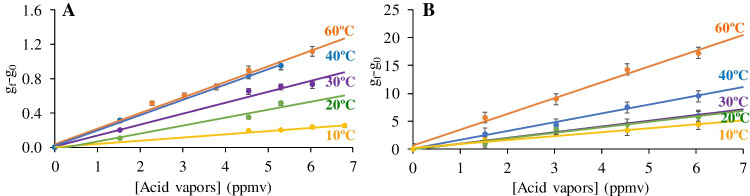
Gray color coordinate (g_f_-g_0_) versus temperature on Color-NSPs. **A** Cellulose-PANI; **B** Nylon-NPs-PANI

The response of Color-NSPs versus temperature and %RH was also evaluated. To do so, the response of the membranes versus acid vapors was evaluated at constant RH (55%) for different temperatures from 10 to 60 °C. As shown in Fig. 3, the response increase with increasing temperature, as expected for an optical sensor [29, 30].

**Fig. 4:**
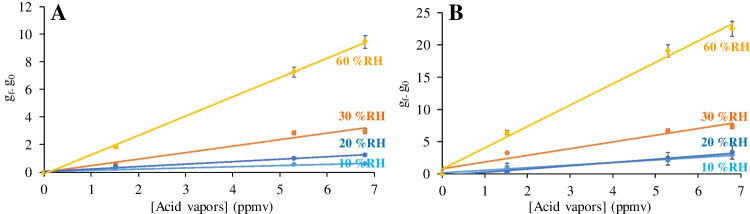
Gray color coordinate (g_f_-g_0_) versus relative humidity on Color-NSPs. **A** Cellulose-PANI; **B** Nylon-NPs-PANI

The influence of %RH in the response of the Color-NSPs was studied in the range from 10 to 60% RH. Figure 4 shows a linear dependence of the analytical response with the RH in the studied range, showing higher sensitivity when the water concentration increase.

To study the influence of gases, both in the range of main atmospheric constituents of O_2_, N_2_, and some contaminants such as SO_2_, HCl, H_2_S, CH_2_O, and CH_4_, the Color-NSPs were exposed to different concentrations of these gases. As expected, the analytical signal of Color-NSPs is only affected by the acidic gases: SO_2_, H_2_S, and HCl.

Table 2 shows a comparison of the Color-NSPs reported here and the recently developed optical gas sensors working on basis of pH indicators. It is observed that the procedure proposed here presents a detection limit close to the rest of the existing methods in the literature, with exposure and recovery times that are within the average values.

**Table 2:** Comparison of performance of the proposed method for acid vapors with literature

Sensing chemistry	Analyte	Technique	Measurement range (ppmv)	Exposure time (s)	Recovery time (s)	LOD (ppmv)	Ref.
MEH-PPV films	HF	PL	0–6000	5	48	0.04	[31]
ZnO/PANI	HAc	PL	1–10	30	300	1.20	[22]
MoS_2_ nanosheet	HF	FO	50–250	150	300	50	[32]
PANI-functionalized nanofibers	HCl	Colorimetric	-	300	60	0.04	[20]
Color-NSPs	HAc	Colorimetric	0–7	300	60	0.95–0.4	Current Study

Poly[2-methoxy-5-(2-ethylhetoxyloxy)-1,4-phenylene (MEH-PPV films); *PL* photoluminescence, *FSP* flame spray pyrolysis, *DRS* diffuse reflectance spectroscopy, *HF* formic acid

### Applications of Color-NSPs for quantification of acid vapors in museums

Cellu-PANI and Nylon-NPs-PANI were applied to the determination of acid vapor concentrations in two different locations from Granada (Spain): “El Fabricante” museum from the Faculty of Fine Arts at the University of Granada (A) and different locations of the Archive of the Royal Chancery of Granada (B).

**Fig. 5:**
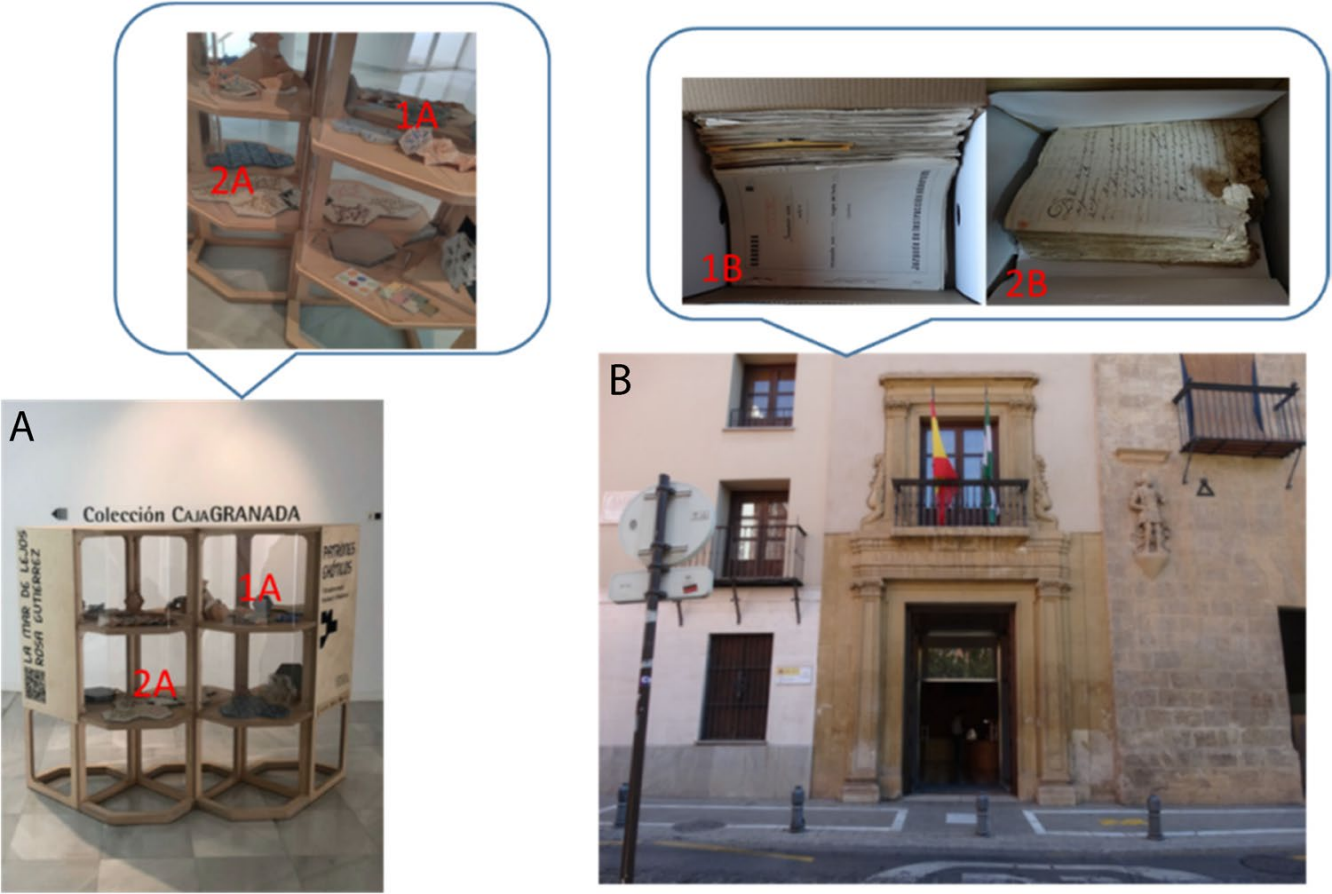
Image of the different locations where the study of the organic acid vapor content was carried out: “El Fabricante” museum (Granada, Spain): 1A books with paper binding 2A tiles of hydraulic cement; and the Archive of the Royal Chancery (Granada, Spain): 1B a drawer containing paper documents and 2B a drawer containing badly damaged paper documents

The Color-NSPs and A-D strip (reference method) were placed in four different locations in the museums: 1A books with paper binding, 2A tiles of hydraulic cement, 1B a drawer containing paper documents, and 2B a drawer containing badly damaged paper documents (see Fig. 5).

Three replicates of Cellu-PANI, Nylon-NPs-PANI, and A-D strips (reference method) were deposited in each selected locations. In all cases, the temperature and humidity conditions were measured and considered to establish the final content of acid vapors. Measurements were taken periodically (twice a week), until no significant change in the analytical signal was observed (see Fig. S7). Table 3 summarizes the results of the concentrations of acid vapors obtained in each of the locations with the proposed methods and those obtained with the reference method. The results obtained with Color-NSPs were compared with those obtained with the reference method by paired test, and *p-*values higher than 5% were obtained, indicating that there are no significant differences between the concentration found with Cellu-PANI, Nylon-NPs-PANI, and the reference method; keeping in mind that the reference method correlates each color with a wide pH range, the pH values obtained with our sensing phases were validated by a paired t-test comparing them with the central value of the pH range obtained with the reference method. As can be seen, the use of Cellu-PANI and Nylon-NPs-PANI offers the possibility of quantifying the concentration of acid vapors in a simple, precise, and fast way.

**Table 3:** Acid vapor concentration comparison by proposed and external reference methods

Sample	Cellu-PANI (ppmv)	Nylon-NPs-PANI (ppmv)	Ref. (ppmv)
1A	4 ± 1 (*n* = 3)	4 ± 1 (*n* = 3)	3–5
2A	1.2 ± 0.2 (*n* = 3)	1.00 ± 0.02 (*n* = 3)	1–2
1B	3 ± 1 (*n* = 3)	2.1 ± 0.2 (*n* = 3)	1–2
2B	4 ± 1 (*n* = 3)	3.3 ± 0.1 (*n* = 3)	3–5

## Conclusions

In this paper, two colorimetric nanostructured sensor phases (Cellu-PANI and Nylon-NPs-PANI) for analyzing the acid vapor concentration in the atmospheres of paper storage rooms have been developed. The developed sensor phases combine nanotechnology (nanoparticles and nanostructured porous membranes; nylon and cellulose) with the detection by color coordinates, which has led to a significant improvement in the accuracy, sensitivity, and response time. Nylon-NPs-PANI shows a lower detection limit, (0.4 ppmv) than Cellu-PANI (0.95 ppmv). This difference may be due to the fact that the combination of nanoparticles with the porous Nylon membrane results in a greater grafted surface area of polyaniline accessible to acid vapors. Cellu-PANI and Nylon-NPs-PANI were used to quantify the acid vapor concentration inside different museum locations. No significant differences between the values obtained with Cellu-PANI and Nylon-NPs-PANI and those obtained with the reference method were found, showing that Cellu-PANI and Nylon-NPs-PANI offer the possibility of quantifying the concentration of acid vapors in a simple, precise, and fast way. Although Nylon-NPs-PANI has a lower detection limit than Cellu-PANI, it should be noted that cellulose membrane is much simpler and easier to manufacture. Both, after a while in open space, stop responding because it suffers saturation, being necessary to regenerate them in the way indicated above in order to continue using them. So for applications where sensitivity is not the determining factor, cellulose membrane would be the ideal candidate. In addition, to our knowledge, it is the first time that acid vapors in the atmospheres of paper storage rooms are accurately quantified; it should be noted that the reference method is semi-quantitative establishing a wide range of concentration by a color chart.

## Supplementary Information

Below is the link to the electronic supplementary material.

Supplementary file1 (DOCX 872 KB)
