# Supplementary material for: Design of colorimetric nanostructured sensor phases for simple and fast quantification of low concentrations of acid vapors
Source: Mikrochim Acta. 2023 Mar 27;190(4):160. doi: 10.1007/s00604-023-05723-0 (PMC10042966; doi:10.1007/s00604-023-05723-0)
Supplement: Supplementary file 1 — Supplementary file1 (DOCX 872 KB) [file 604_2023_5723_MOESM1_ESM.docx]

**Electronic Supplementary Material**

**Design of Colorimetric Nanostructured Sensor Phases for simple and fast quantification of low concentrations of Acid Vapors**

M.D. Fernández-Ramos^*a,b^, M. Bastida Armesto^a^, R. Blanc-García^c^, L.F. Capitán-Vallvey^a,b^ and A.L. Medina-Castillo* ^a,b^

*^a^ECsens. Department of Analytical Chemistry, University of Granada, Granada 18071 (Spain).*

*^b^Unit of Excellence in Chemistry applied to Biomedicine and the Environment of the University of Granada.*

*^c^Department of Analytical Chemistry, University of Granada, Granada 18071 (Spain)*

*Corresponding author:^*^e-mail:* [*mdframos@ugr.es*](mailto:mdframos@ugr.es)*; ^*^email: antonioluismedina@ugr.es*

**INDEX**

**Fig. S1.** Image of measurement system.

**Fig. S2** and **Fig. S3.** Selection of the Colour coordinate.

**Fig. S4**. Color-NSPs exposure time to acid vapor.

**Fig. S5.** Calibrations plots

**Fig. S6.** Long-term stability of Color-NSPs

**Fig. S7.** Variation of colour coordinates versus time in location 1A.

**Fig. S8.** Dynamic response of Colour-NSPs at location 1A.

***
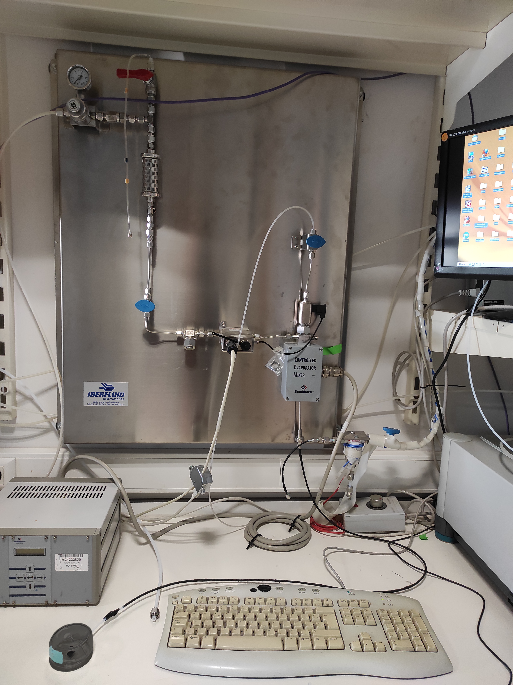
***

**Fig. S1.** Image of Controlled Evaporator Mixer system (CEM) and homemade climate chamber with the Color-NSPs inside.

**Fig. S2.** Colour coordinates versus concentration of acids vapour: RGB Cellulose-PANI; (A1), and Nylon-NPs-PANI; (B1). HSV for Cellulose-PANI; (A2) and Nylon-NPs-PANI; (B2).

**Fig. S3.** Gray colour coordinate versus concentration of acid vapours of: Cellulose-PANI; A and Nylon-NPs-PANI; B.


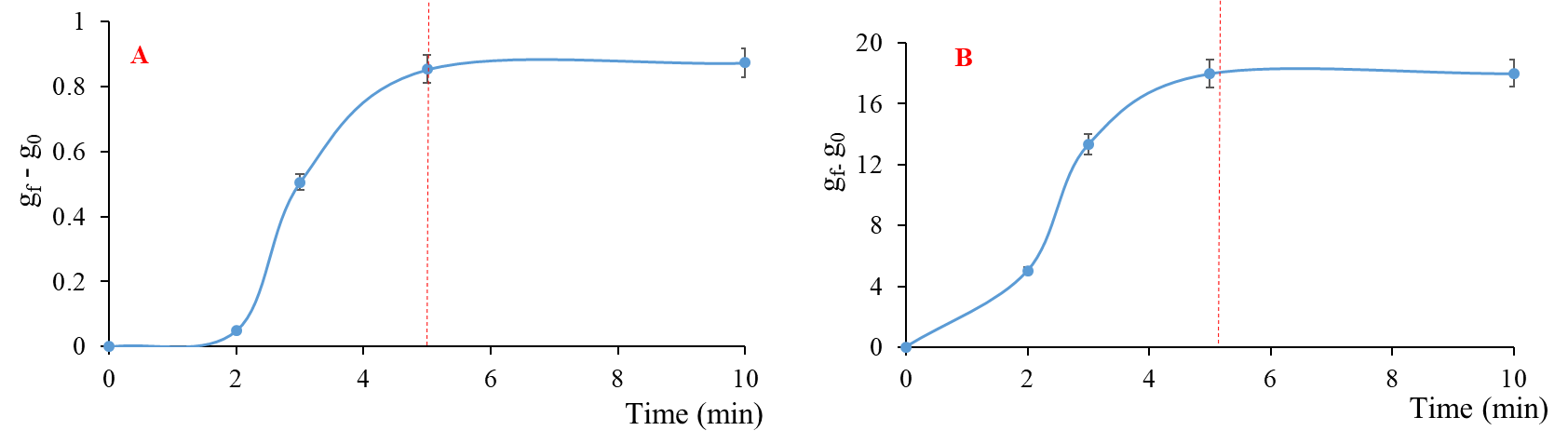


**Fig. S4.** Gray colour coordinate versus exposure time to acid vapours: Cellulose-PANI; A and Nylon-NPs-PANI; B.

**Fig. S5.** Calibration plots: A: Cellu-PANI; B: Nylon-NPs-PANI from 1 to 7 ppmv acid vapors, generated from a 3 mM aqueous solution of acetic acid varying the amount of vapor from 0.3 to 3 g·h-1 in a dry air current of 5 ln·min-1

**Fig. S6**. Shewhart control chart for check the long-term stability: Cellulose-PANI; A and Nylon-NPs-PANI; B.


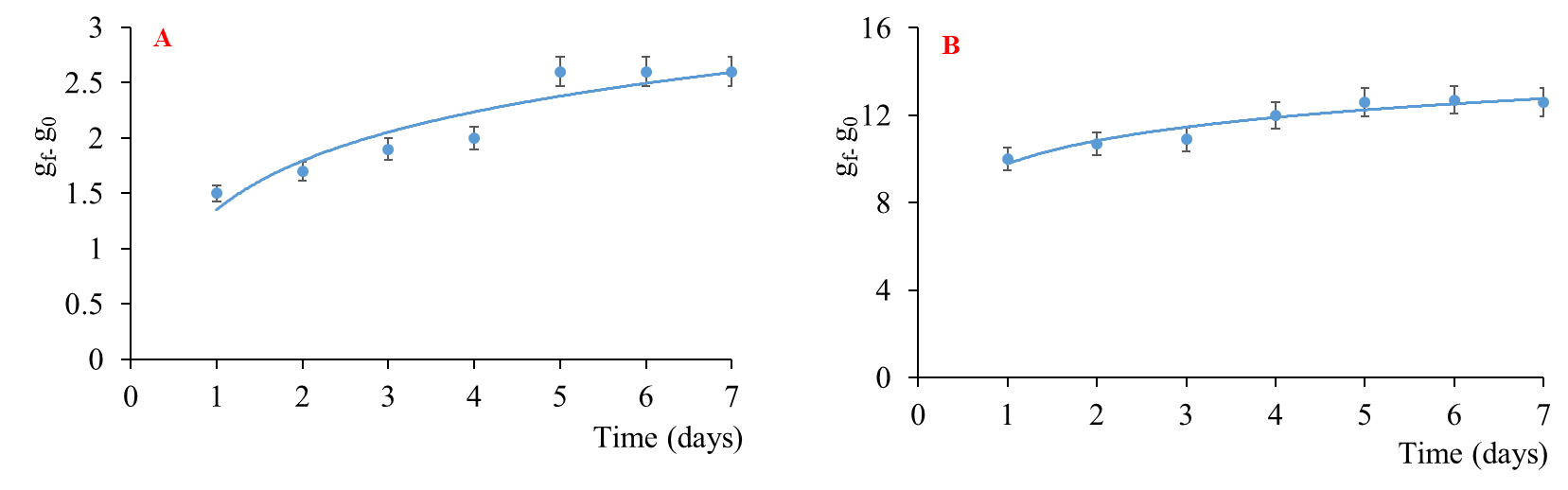


**Fig. S7.** Dynamic response of Color-NSPs at location 1A: Cellulose-PANI; A, Nylon-

NPs-PANI; B.
